# Usual medical treatments or levonorgestrel-IUS for women with heavy menstrual bleeding: long-term randomised pragmatic trial in primary care

**DOI:** 10.3399/bjgp16X687577

**Published:** 2016-10-11

**Authors:** Joe Kai, Lee Middleton, Jane Daniels, Helen Pattison, Konstantinos Tryposkiadis, Janesh Gupta

**Affiliations:** Faculty of Medicine & Health Sciences, School of Medicine, University of Nottingham, Nottingham.; Birmingham Clinical Trials Unit, University of Birmingham, Birmingham.; Birmingham Clinical Trials Unit, University of Birmingham, Birmingham.; School of Life and Health Sciences, Aston University, Birmingham.; Birmingham Clinical Trials Unit, University of Birmingham, Birmingham.; Birmingham Women’s Foundation NHS Trust, Birmingham.

**Keywords:** general practice, levonorgestrel intrauterine system, medical treatment, menorrhagia, menstrual, primary health care

## Abstract

**Background:**

Heavy menstrual bleeding (HMB) is a common, chronic problem affecting women and health services. However, long-term evidence on treatment in primary care is lacking.

**Aim:**

To assess the effectiveness of commencing the levonorgestrel-releasing intrauterine system (LNG-IUS) or usual medical treatments for women presenting with HMB in general practice.

**Design and setting:**

A pragmatic, multicentre, parallel, open-label, long term, randomised controlled trial in 63 primary care practices across the English Midlands.

**Method:**

In total, 571 women aged 25–50 years, with HMB were randomised to LNG-IUS or usual medical treatment (tranexamic/mefenamic acid, combined oestrogen–progestogen, or progesterone alone). The primary outcome was the patient reported Menorrhagia Multi-Attribute Scale (MMAS, measuring effect of HMB on practical difficulties, social life, psychological and physical health, and work and family life; scores from 0 to 100). Secondary outcomes included surgical intervention (endometrial ablation/hysterectomy), general quality of life, sexual activity, and safety.

**Results:**

At 5 years post-randomisation, 424 (74%) women provided data. While the difference between LNG-IUS and usual treatment groups was not significant (3.9 points; 95% confidence interval = −0.6 to 8.3; *P* = 0.09), MMAS scores improved significantly in both groups from baseline (mean increase, 44.9 and 43.4 points, respectively; *P*<0.001 for both comparisons). Rates of surgical intervention were low in both groups (surgery-free survival was 80% and 77%; hazard ratio 0.90; 95% CI = 0.62 to 1.31; *P* = 0.6). There was no difference in generic quality of life, sexual activity scores, or serious adverse events.

**Conclusion:**

Large improvements in symptom relief across both groups show treatment for HMB can be successfully initiated with long-term benefit and with only modest need for surgery.

## INTRODUCTION

Heavy menstrual bleeding (HMB) is a common and debilitating problem that can significantly affect the lives of women. With an annual community incidence of 25% among women aged 18–54 years,^1^ 1 million seek help for this problem each year in the UK,^2^ mostly in general practice,^3^ and it accounts for 12% of all gynaecology referrals.^4^ Despite the many factors influencing decisions not to seek help,^5^ the cost of health care for HMB is substantial. In a recent national audit of care of HMB in England and Wales, almost one-third of women had received no previous medical treatment before referral to secondary care, with over 40% having surgical intervention in the year following first attendance at hospital.^6^

The National Institute for Health and Care Excellence (NICE) defines HMB as that which interferes with a women’s physical, emotional, social, and material quality of life, and which can occur alone or with other symptoms.^2^ This recognises that patient perceptions of HMB and what they find troublesome does not correlate well with a traditional biomedical focus on volume of blood loss.^7^ For clinical practice, HMB should be regarded as more subjectively defined by patients, focusing on perceived impact on their lives rather than menstrual blood loss in itself;^2^^,^^8^ in addition to physical and psychological health, this includes interference with social, working, and family life, or the practical burden of sanitary care.^9^

At the commencement of the current trial, guidelines for HMB recommended that initial management should usually be medical, using either oral tranexamic acid or mefenamic acid; or using the combined oral contraceptive (COC), or the levonorgestrel-releasing intrauterine system (LNG-IUS) for women requiring contraception, or for those not requiring contraception but prepared to accept hormonal treatments.^10^ In 2007, similar guidelines from NICE recommended this range of pharmaceutical treatments, and underlined the potential for more patients with HMB to be managed by their GPs, avoiding secondary care.^2^

Five trials in gynaecological settings (involving 44–165 women, and 3–12 months follow-up) have found LNG-IUS more beneficial in reducing menstrual blood loss than treatments such as mefenamic acid or COC.^11^ However, evidence on how helpful treatments are in improving patients’ quality of life, or their use in primary care, is lacking.^11^ Previous findings from the current trial found that both LNG-IUS and usual medical treatments significantly reduced the effect of HMB on patients’ quality of life in the first 2 years of treatment, but LNG-IUS was more effective.^12^ HMB may, however, be chronic and episodic over several years.^13^ A recent Cochrane Review recommended that trials of at least 5 years are needed, which should include a focus on patients’ quality of life.^11^

**How this fits in**

Heavy menstrual bleeding is a chronic debilitating problem, and a common cause of gynaecological referral and surgery. Evidence is lacking about the long-term effectiveness of treatment in primary care. This trial shows that initiation of either the usual medical treatments or the levonorgestrel-releasing intrauterine system in general practice can safely help. These treatments reduce the effects of heavy menstrual bleeding on patients’ lives over a 5-year time course with most women avoiding surgery.

Decisions made by women and their GPs about medical treatments for HMB also include wider dynamic considerations such as attitudes to using oral treatment or having an intrauterine device, changing plans about wanting to conceive or need for contraception, or anticipating surgical intervention. Thus, long-term evidence is needed to help guide decision making in practice.

In this pragmatic randomised controlled trial, outcomes at 5 years of commencing LNG-IUS or usual medical treatments were assessed for women presenting with HMB in primary care.

## METHOD

### Population

Women aged 25–50 years with HMB involving at least three consecutive menstrual cycles and who presented to their GP were eligible to participate. Women were excluded if they intended to become pregnant over the next 5 years, were taking hormone-replacement therapy or tamoxifen, had intermenstrual bleeding (between expected periods), postcoital bleeding, or findings suggestive of fibroids (abdominally-palpable uterus equivalent in size to that at 10–12 weeks’ gestation), or other disorders, or had contraindications to or a preference for either the levonorgestrel-IUS or usual medical treatments. Women with heavy, irregular bleeding were ineligible unless the results of endometrial biopsy were reported to be normal; no further investigations were mandated by the protocol. All patients provided written informed consent.

### Randomisation

Patients were assigned to a study group by telephone or a web-based central randomisation service at the University of Birmingham Clinical Trials Unit. A computerised, minimised randomisation procedure was used to achieve balance between the groups with respect to age (<35 years or ≥35 years), body-mass index (BMI kg/m^2^; <25 or ≥25), duration of symptoms (<1 year or ≥1 year), need for contraception (yes or no), and HMB alone or HMB accompanied by menstrual pain.

### Study interventions and compliance

Eligible women who provided written informed consent were randomly assigned to either LNG-IUS or usual medical treatment. Usual treatment options included oral tranexamic acid, mefenamic acid, norethisterone, a combined oestrogen–progestogen or progesterone-only oral contraceptive pill (any formulation), or medroxyprogesterone acetate injection; and were chosen by the clinician and patient on the basis of any contraceptive needs or the desire to avoid hormonal treatment.^2^^,^^14^ The particular medical treatment to be used was specified before randomisation. Subsequently, and in line with real-life practice, treatments could be changed (from one medical treatment to another, from the LNG-IUS to a usual medical treatment, or from a usual medical treatment to the LNG-IUS), or could be discontinued because of a perceived lack of benefit, side effects, a change in the need for contraception, referral for endometrial ablation or hysterectomy, or any other reasons according to usual clinical practice.^2^^,^^14^ Treatment changes reported by patients were confirmed with their GP.

### Outcome measures and follow-up

The primary outcome measure was the patient-reported, condition-specific Menorrhagia Multi-Attribute Scale (MMAS) at 5 years follow-up.^15^^,^^16^ The MMAS is designed to measure the effect of HMB on six domains of daily life. Possible responses are not affected/slightly affected/moderately affected/severely affected for each domain. The scores for each domain are weighted according to the perceived importance of that domain to women with this condition. In order of importance, from highest to lowest, the domains are: family life and relationships; physical health; work and daily routine; practical difficulties; psychological health; social life. Summary scores, which range from 0 (severely affected) to 100 (not affected), were assessed. The MMAS has a high degree of reliability and internal consistency,^15^ good content and construct validity,^17^^,^^18^ is responsive,^19^^,^^20^ and is acceptable to responders.^15^^,^^16^^,^^19^^,^^20^

Secondary outcome measures included general health-related quality of life and sexual activity. To assess generic quality of life, three instruments were used: the Medical Outcomes Study 36-Item Short-Form Health Survey (SF-36), version 2 (with scores ranging from 0 [severely affected] to 100 [not affected]); the EuroQoL Group 5-Dimension Self-Report Questionnaire (EQ-5D) descriptive system (with scores ranging from −0.59 [health state worse than death] to 100 [perfect health state]); and the EQ-5D visual-analogue scale (with scores ranging from 0 [worst health state imaginable] to 100 [most perfect health state imaginable]). The validated Sexual Activity Questionnaire measures pleasure (with scores ranging from 0 [lowest level] to 18 [highest level]), discomfort (with scores ranging from 0 [greatest] to 6 [none]), and frequency (assessed relative to perceived usual activity as an ordinal response).^21^ Responses for all outcomes were obtained before randomisation and by post at 5 years after randomisation. Data were collected from participating GPs regarding all serious adverse events, defined as adverse events that resulted in death, disability, or hospitalisation. Patients were also asked to report any hospitalisations and adverse events leading to discontinuation of the study treatments.

### Study oversight

Study oversight was provided by an independent steering committee and an independent data and safety monitoring committee, whose three reviews of interim data provided no reason to modify the trial protocol on the basis of pragmatic stopping criteria.^22^ The study was conducted in accordance with the protocol, available at www.nets.nihr.ac.uk/projects/hta/020602. All medications and devices were prescribed by providers through the NHSconfid. The manufacturers of any therapeutic agents used in the study were not involved in any aspect of the trial.

### Statistical considerations

The study was designed to have 90% power (at *P*<0.05) to detect a small-to-moderate difference^23^ — 0.3 of a standard deviation (SD) — in the primary outcome. This required responses from 470 patients; the sample size was increased to 570 to allow for up to 20% loss to follow-up. At 5 years follow-up 424 responses were received; a post-hoc calculation suggested that this total would provide 87% power (*P* = 0.05) to detect the same size of difference. For progression to surgical intervention (hysterectomy or endometrial ablation), using an assumed rate of 35% in the standard arm (a figure that was set out in the protocol), 424 women would provide 80% power (*P* = 0.05) to detect an absolute reduction of 12%, that is 35% down to 23%.

Analyses were performed according to the intention-to-treat principle. Differences between groups at 5 years were examined by analysis of covariance (adjusting for baseline score). Changes between baseline score within groups were examined using paired *t*-tests. The primary analysis was based on some patients declining to complete the MMAS, indicating on the form that they were no longer bleeding and the questions did not appear relevant to them. Thus, these patients were assigned the best possible score (100). This assumption was further tested through sensitivity analyses by making no assumption about those questionnaires that were returned blank, that is MMAS scores were assumed to be missing. Kaplan–Meier plots were constructed for a time to surgery and a time to treatment change analysis, with women censored at date to last followup or, if appropriate, date to death, withdrawal or loss to follow-up. A Cox proportional hazards model was used to construct hazard ratios (HRs). Surgery-free analysis was then re-performed, excluding participants who crossed over from one treatment group to another. All the effect sizes are presented with 95% confidence intervals (CIs) and *P*-values. All tests and corresponding *P*-values were two-sided. The statistical package SAS (version 9.2) was used for all the statistical analysis.

## RESULTS

### Patients and follow-up

Between February 2005 and July 2009, a total of 571 women with HMB from 63 primary care centres were randomly assigned to either the LNG-IUS (285 women) or usual medical treatment (286 women). Baseline characteristics were similar between the two treatment groups (Table 1).

**Table 1.** Characteristics of the patients

		**Usual medical treatment, *n* (%)**	**LNG-IUS, *n* (%)**
*N*		286	285

Age, years	≥35^a^	255 (89)	257 (90)
	Mean (SD)	41.8 (5.5)	42.1 (5.0)

Body mass index, kg/m^2^	≥25^a^	200 (70)	200 (70)
	Mean (SD)	29.3 (6.7)	29.1 (6.1)

Ethnic group^b^	White	246 (86)	225 (79)
	Asian	23 (8)	28 (10)
	Black	12 (4)	18 (6)
	Mixed	4 (1)	9 (3)
	Other	1 (<1)	4 (1)

Duration of heavy menstrual bleeding ≥1 year^a^		229 (80)	231 (81)

Presence of menstrual pain^a^		211 (74)	213 (75)

Contraceptive requirement^a^		55 (19)	55 (19)

Copper or non-hormonal coil in place		10 (3)	9 (3)

LNG-IUS = levonorgestrel-releasing intrauterine system.

a This characteristic was a minimisation variable and was assessed in the pre-defined subgroup analyses.

b Self-identified ethnic grouping; one ‘not given’ response in the LNG-IUS group.

For 215 (75%) of the women assigned to usual medical treatment, the initial prescription was for mefenamic acid, tranexamic acid, or a combination of the two drugs; 55 (19%) of the women in the usual treatment group required contraception.

**Figure 1.**
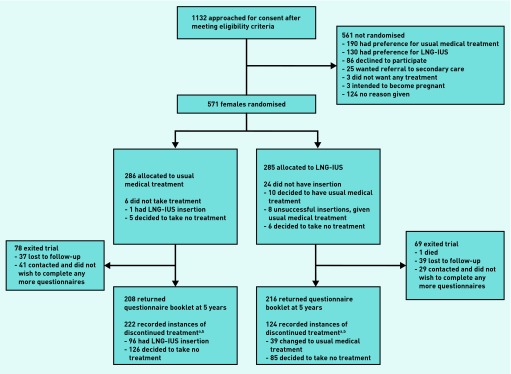
***Enrolment, randomisation and follow-up for up to 5 years of the study patients. ^a^See Figure 2 for time to first treatment change survival estimates. ^b^Reasons reported for discontinuing treatment available from the authors on request.***

Study-questionnaire booklets were returned by 424 (74%) of participants at 5 years (Figure 1). One-hundred and fifteen women (27%) indicated that they were no longer having periods and so did not complete the MMAS section.

These women completed other sections of the questionnaire and still contributed to the analysis of MMAS responses.

**Figure 2.**
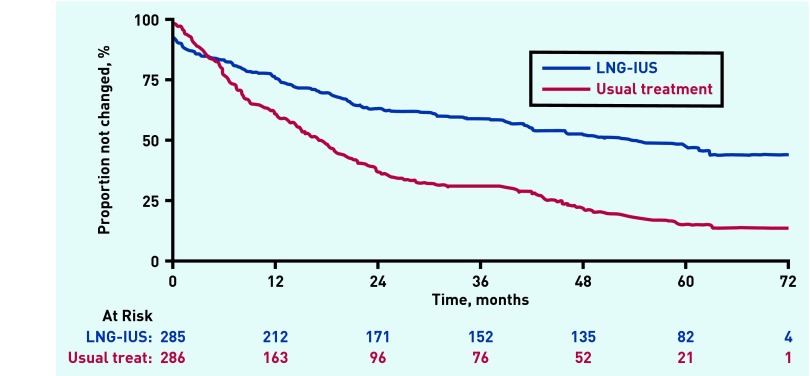
***Time to first treatment change over 5 years follow-up.***

The proportion of patients still taking their allocated treatment at 5 years was 47% (95% CI = 40% to 52%) in the LNG-IUS group and 15% (95% CI = 11% to 20%) in the usual treatment group (Figure 2).

Of the 228 recorded instances of treatment change in women allocated usual medical treatment, 97 (43%) were to LNG-IUS. In the LNG-IUS group, 57 out of 148 (39%) treatment switches were to usual treatment.

Reported reasons for discontinuation of treatment were varied, with lack of treatment efficacy most commonly cited (24% [36 out of 148] in the LNG-IUS group and 41% [94 out of 228] in the usual treatment group).

Further details are summarised in Appendices 1 and 2 (available from authors on request).

### Primary outcome: Menorrhagia Multi-Attribute Scale (MMAS)

Women started out with average scores of approximately 40 points out of 100 on the MMAS, indicating that they were substantially affected by HMB at presentation to their GP.

At 5 years, these scores were significantly improved to >80 points out of 100 in both groups (Table 2).

**Table 2.** Menorrhagia Multi-Attribute Scale (MMAS) summary scores at baseline and 5 years follow-up

**Baseline score (SD, *n*)**		**5 year score (SD, *n*)**		**Difference between groups over 5 years (95%CI) ^a^, *P*-value**	**Change within group (95% CI), *P*-value**	
		
**UMT**	**LNG-IUS**	**UMT**	**LNG-IUS**		**UMT**	**LNG-IUS**
39.2 (21.3, 269)	42.5 (20.5, 280)	83.1 (24.4, 208)	87.1 (22.1, 216)	3.9 (−0.6 to 8.3), 0.09	43.9 (39.1 to 47.7), <0.0001	44.6 (41.0 to 48.8), <0.0001

Mean score (standard deviation, n) shown at each time-point.

a Point estimates (95% CI, P-values) shown for differences between groups and changes within group. Estimates >0 from differences between groups favour LNG-IUS. Results at each time-point adjusted for baseline score. Change within group compared to baseline. 0 = worst affected, 100 = not affected. LNG-IUS = levonorgestrel-releasing intrauterine system. UMT = usual medical treatment.

This improvement was higher on average among the women assigned to LNG-IUS but the difference was not statistically significant (3.9 points; 95% CI = −0.6 to 8.3; *P* = 0.09). The same analysis without any assumption about MMAS scores, where the form was returned blank and the women indicated she was no longer bleeding, returned a similar result (5.2 points difference in favour of LNG-IUS; 95% CI = −0.4 to 10.8; *P* = 0.07).

### Surgical interventions

**Figure 3.**
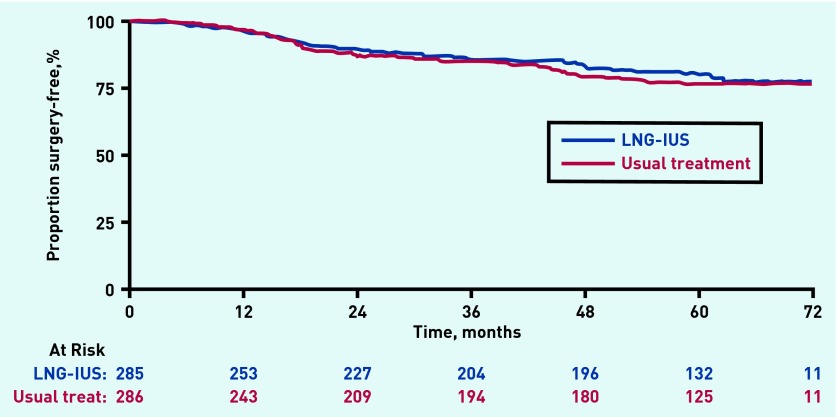
***Surgery-free (hysterectomy/endometrial ablation) survival analysis over 5 years of follow-up.***

Fifty-three events (endometrial ablation or hysterectomy) in the LNG-IUS group versus 56 in the usual treatment group were included in the surgery-free survival analysis (109 events in total). This difference was not statistically significant (HR 0.90; 95% CI = 0.62 to 1.31; *P* = 0.6) (Figure 3). Analysis excluding participants who crossed over from one group to another returned a similar result (HR 0.96; 95% CI = 0.60 to 1.52; *P* = 0.9). Five-year surgery-free survival rates were 80% (95% CI = 74% to 84%) in the LNG-IUS group versus 77% (95% CI = 71% to 82%) in the usual treatment group. In total, there were 115 surgical interventions: 24 ablations in the LNG-IUS group versus 31 in the usual treatment group and 30 hysterectomies in both groups (six more events than quoted above as six patients had both types of surgery).

### Generic quality of life and sexual activity

Responses to the EuroQoL and SF-36 instruments were generally significantly improved from baseline in both groups (Table 3); the only statistically significant difference between groups was seen in the general health perception domain of the SF-36 and favoured LNG-IUS (4.7 points, 95% CI = 0.6 points to 8.8 points; *P* = 0.02). The treatment groups did not differ significantly with respect to any of the domains of the Sexual Activity Questionnaire.

**Table 3.** Scores on the quality-of-life and sexual activity questionnaires at baseline and 5 years follow-up

	**Baseline score (SD, *n*)**		**5 year score (SD, *n*)**		**Difference between groups over 5 years (95% CI)^a^, *P*-value**	**Change within group (95 % CI), *P*-value**	
		
	**UMT**	**LNG-IUS**	**UMT**	**LNG-IUS**		**UMT**	**LNG-IUS**
**SF-36^b^**							
Physical functioning	77.8 (24.7, 264)	80.0 (20.4, 272)	83.0 (26.3, 208)	85.4 (23.5, 216)	1.6 (−2.7 to 5.9), 0.5000	5.6 (1.8 to 9.5), 0.0040	5.9 (2.8 to 9.1), 0.0003
Physical role	68.9 (26.2, 264)	72.1 (24.7, 276)	80.6 (27.2, 208)	83.9 (25.5, 217)	2.7 (−2.1 to 7.5), 0.3000	12.6 (7.9 to 17.2), <0.0001	13.3 (9.5 to 17.1) <0.0001
Emotional role	69.8 (26.8, 264)	71.9 (25.1, 276)	83.3 (25.3, 208)	81.4 (26.9, 217)	−2.0 (−6.8 to 2.9), 0.4000	13.1 (8.7 to 17.6), <0.0001	10.9 (6.7 to 15.1), <0.0001
Social functioning	62.4 (25.9, 268)	64.3 (24.5, 277)	76.9 (25.5, 207)	79.5 (25.1, 214)	2.2 (−2.5 to 6.9), 0.4000	14.0 (9.4 to 18.5), <0.0001	15.6 (11.6 to 19.6), <0.0001
Mental health	59.0 (19.8, 267)	60.3 (19.3, 277)	72.6 (19.4, 206)	71.4 (20.5, 214)	−1.6 (−5.2 to 2.0), 0.4000	13.6 (10.5 to 16.7), <0.0001	11.4 (8.5 to 14.3), <0.0001
Energy/vitality	40.8 (21.7, 268)	40.7 (20.9, 277)	56.6 (23.1, 206)	59.2 (21.0, 213)	2.8 (−1.2 to 6.9), 0.2000	15.7 (11.9 to 19.4), <0.0001	18.6 (15.2 to 22.0), <0.0001
Pain	49.5 (24.9, 268)	54.2 (24.9, 278)	71.3 (28.6, 207)	76.1 (24.8, 214)	3.7 (−1.3 to 8.7), 0.1000	21.4 (16.9 to 25.8), <0.0001	22.7 (18.6 to 26.9), <0.0001
General health perception	60.3 (21.9, 264)	61.8 (21.4, 274)	61.9 (23.8, 207)	67.8 (22.1, 214)	4.7 (0.6 to 8.8), 0.0200	2.0 (−1.5 to 5.6), 0.2600	5.1 (2.0 to 8.3), 0.0020

**EuroQoL**							
EQ-5D descriptive system^c^	0.71 (0.28, 269)	0.76 (0.24, 277)	0.82 (0.25, 207)	0.81 (0.25, 214)	−0.02 (−0.06 to 0.02), 0.4000	0.10 (0.06 to 0.14), <0.0001	0.06 (0.02 to 0.09), 0.0030
EQ-5D visual analogue scale^d^	69.7 (19.8, 246)	70.3 (19.1, 250)	76.1 (18.8, 198)	77.3 (19.9, 207)	0.6 (−3.2 to 4.5), 0.8000	6.3 (2.9 to 9.8), 0.0004	6.5 (3.0 to 9.9), 0.0003

**Sexual activity questionnaire^e^**							
Pleasure	10.9 (4.9, 199)	10.8 (4.9, 210)	11.8 (4.6, 100)	11.2 (4.5, 111)	−0.4 (−1.7 to 0.9), 0.6000	0.6 (−0.6 to 1.7), 0.4000	0.1 (−1.0 to 1.2), 0.9000
Discomfort	4.6 (1.7, 201)	4.7 (1.5, 209)	4.8 (1.6, 101)	4.8 (1.6, 111)	0.0 (−0.4 to 0.4), 0.9000	0.1 (−0.2 to 0.4), 0.7000	0.1 (−0.3 to 0.4), 0.7000

Mean score (standard deviation, n) shown at each time-point. Point estimates (95% CI, P-values) shown for differences between groups and changes within group. Estimates >0 from differences between groups favour LNG-IUS. Results at each time-point adjusted for baseline score. Change within group compared with baseline.

a Estimated values >0 favour LNG-IUS.

b The Medical Outcomes Study 36-Item Short-Form Health Survey (SF-36) is a general health-related quality-of-life questionnaire. Scores in each of the eight domains range from 0 (severely affected) to 100 (not affected).

c The EuroQoL Group 5-Dimension Self-Report Questionnaire (EQ-5D) descriptive system is a general health-related quality-of-life questionnaire. Scores range from −0.59 (state of health worse than death) to 1.00 (perfect state of health).

d Scores on the EQ-5D visual-analogue scale range from 0 (worst health state imaginable) to 100 (most perfect health state imaginable).

e The Sexual Activity Questionnaire is designed to assess the possible effect of treatment on sexual functioning. Scores for pleasure range from 0 (lowest level) to 18 (highest level), and scores for discomfort range from 0 (greatest) to 6 (none). LNG-IUS = levonorgestrel-releasing intrauterine system. UMT = usual medical treatment.

### Safety

There was no significant difference between the groups in the total number of serious adverse events (*P* = 0.32). These are listed in Appendix 2 (available from authors on request).

## DISCUSSION

### Summary

This pragmatic trial shows that women affected by HMB can be effectively helped in primary care by initiating LNG-IUS or usual medical treatment, with long-term benefit, and only modest need for surgery. Women in either treatment group experienced similar and significant improvement in condition-specific quality of life after 5 years. Women receiving usual medical treatment were no more likely to need surgical intervention than those treated with insertion of an LNG-IUS, with rates of surgical intervention (endometrial ablation or hysterectomy) remaining low in both groups (approximately 20%). Generic quality of life scores were similarly improved in both groups and there was no difference in sexual activity scores or serious adverse events.

### Strengths and limitations

This is the largest randomised trial available of medical treatments for HMB. Generalisability is strengthened by a pragmatic, multicentre design, mimicking treatment decisions in ‘real life’ primary care, involving a large sample ethnically representative of the UK population. Outcomes have been assessed in the longer term, appropriate to the chronic nature of HMB. This study used a validated patient-centred primary outcome reflecting patients’ assessments of the impact of HMB on their quality of life, in line with guidance for assessing HMB,^2^ rather than a biomedical focus on menstrual blood loss itself. While use of indirect measures of menstrual blood loss were considered, pictorial blood assessment charts correlate poorly with blood loss and are not consistently accurate.^8^

Given 5 years since study entry, a relatively high follow-up has been sustained to include 424 out of 571 (74%) women randomised. The range of drugs within the usual medical treatment group includes those used in routine practice, but it is acknowledged that this limits ability to compare any individually with the LNG-IUS. The intention-to-treat analysis may be considered overly conservative by some; particularly given the long follow-up period but alternatives such as per protocol analyses are likely to exaggerate treatment effects as they restrict analyses to only those patients satisfied with treatment performance.^24^ While there was no statistically significant difference in primary outcome between the two groups, significant proportions of women reported that their periods had ceased, or had changed or ceased treatments, and this may have limited the ability to detect a difference. Such changes may be expected in real life over 5 years, however, and are consistent with experience from national audit of care for HMB.^6^

### Comparison with existing literature

A 2015 Cochrane Review highlights the lack of research on the medical management of HMB in primary care, the need for evidence on HMB related quality of life outcomes, and for data from longer-term trials reporting beyond 2 years.^11^ The current trial contributes new evidence to these three gaps. The authors are not aware of similar long-term comparisons of medical treatments initiated in primary care. In secondary care, trials of similar length have compared LNG-IUS with hysterectomy^25^ rather than other medical treatments, or endometrial resection to oral medication.^26^

The present results are encouraging in showing both HMB-specific and generic quality of life for women were significantly improved 5 years after commencing either usual medical treatment or LNG-IUS. The size of improvement — approximately equivalent to two SDs — is likely to be a very large effect.^23^ Women were considerably affected by HMB at presentation to their GP, and improvement in MMAS score from baseline (by 43.9 and 44.6 points for usual medical treatment or for LNG-IUS, respectively) reflects a clinical change of at least one category in all six MMAS domains (practical difficulties, social life, psychological health, physical health, work and daily routine, and family life and relationships) from being substantially to minimally affected by HMB; for example, from frequent to occasional disruptions of work and daily routine.

The greater clinical efficacy of LNG-IUS compared with usual medical treatments seen at 2 years in this trial^12^ has now diminished. At 5 years, only a borderline difference (*P* = 0.09) in favour of LNG-IUS has been observed. This was estimated to be 3.9 points on average, which is less than one-fifth of a SD and unlikely to be clinically meaningful. This may be unsurprising given the high proportions of women who, by 5 years after treatment allocation, had either changed to a treatment that worked for them, or had ceased bleeding either naturally or through surgical intervention. Retention rates at 5 years were 15% with usual medical treatment and 47% in the LNG-IUS group. This may reflect greater impact on symptoms from using the LNG-IUS. Another factor may be that women could more easily choose to stop usual medical treatment when desired or according to their symptoms, without the need for consultation and removal of their intra-uterine device (IUD).

Natural cessation of bleeding or women stopping treatments may also explain the present data providing no evidence of any reduction in surgical interventions with LNG-IUS compared with usual medical therapy, even when treatment cross-overs were discounted. At 2 years post-randomisation, surgical interventions were low at about 10% in both medical treatment groups,^12^ and this has approximately doubled to 20% in both groups at 5 years. This is still much lower than the 58% surgical intervention rate at 2 years identified in an earlier Cochrane Review of trials comparing oral medical therapy with surgical interventions for HMB, although these were in secondary care settings.^27^ The relatively low surgical intervention rates in the current trial may also possibly be explained by exclusion of women with enlarged uteri or known conditions such as fibroids that were deemed unsuitable for treatment in a community setting.

### Implications for research and practice

The present data have been obtained in the context of real-life clinical practice, for a chronic and episodic problem, where, as experienced in this trial, women may commonly discontinue or change treatments for their HMB.^6^ The inclusion criteria also underline that women who do not have a uterus palpable abdominally, or who have had normal investigation for irregular periods, can be successfully treated in primary care.

The results provide valuable practical information for women and GPs when weighing up choice of, and what to expect in the longer term from treatments for HMB. This needs to take account of individuals differing preferences for oral treatments or insertion of an IUD left in situ and changing needs for contraception or fertility.^28^ The study shows that women can benefit significantly from choosing either usual medical or LNG-IUS treatment. Just under half of women may be expected to retain their LNG-IUS at 5 years, while most have ceased usual oral treatments by this stage. Women able to choose LNG-IUS, if suited to their circumstances, may experience less discontinuation of treatment, and a better effect at 2 years.^12^ This will not suit all women however, as 36% in the current trial had had their LNG-IUS removed by 2 years because of persisting HMB or unpredictability of bleeding^12^ and this is a well-recognised problem.^29^

The low rates of progression to surgical intervention observed, 5 years from initial presentation with HMB to their GP, emphasise the feasibility and importance of treating women with HMB in primary care. Avoiding referrals to secondary care may reduce high operative intervention rates.^6^^,^^30^

Wider public awareness is needed to encourage women to seek help for HMB as they are likely to benefit from LNG-IUS or usual medical treatment in primary care. Commensurate availability of expertise to offer this range of medical treatments should be ensured. While the present data suggest that the earlier superiority of LNG-IUS over the first 2 years was not sustained at 5 years, further research to confirm this by assessing patient satisfaction with and acceptability of treatments would be helpful. Longitudinal qualitative research is needed to explore and understand women’s decisions in choosing treatments for HMB and experiences of using them over time. The present authors intend to follow-up patients to 10 years, when it is expected that around half of the cohort will have reached the menopause, to assess further patterns of treatment use and surgical intervention rates.

This pragmatic trial confirms that women affected by HMB, with no significant clinical risk factors on history or examination, can be safely helped by initiating medical treatments in primary care, with long-term benefit in reducing the effects of HMB on their quality of life.
